# Cauchy combination omnibus test for normality

**DOI:** 10.1371/journal.pone.0289498

**Published:** 2023-08-03

**Authors:** Zhen Meng, Zhenzhen Jiang

**Affiliations:** 1 School of Statistics, Capital University of Economics and Business, Beijing, China; 2 Academy of Mathematics and Systems Science, Chinese Academy of Sciences, Beijing, China; 3 School of Mathematical Sciences, University of Chinese Academy of Sciences, Beijing, China; Icahn School of Medicine at Mount Sinai, UNITED STATES

## Abstract

Testing whether data are from a normal distribution is a traditional problem and is of great concern for data analyses. The normality is the premise of many statistical methods, such as *t*-test, Hotelling *T*^2^ test and ANOVA. There are numerous tests in the literature and the commonly used ones are Anderson-Darling test, Shapiro-Wilk test and Jarque-Bera test. Each test has its own advantageous points since they are developed for specific patterns and there is no method that consistently performs optimally in all situations. Since the data distribution of practical problems can be complex and diverse, we propose a Cauchy Combination Omnibus Test (CCOT) that is robust and valid in most data cases. We also give some theoretical results to analyze the good properties of CCOT. Two obvious advantages of CCOT are that not only does CCOT have a display expression for calculating statistical significance, but extensive simulation results show its robustness regardless of the shape of distribution the data comes from. Applications to South African Heart Disease and Neonatal Hearing Impairment data further illustrate its practicability.

## Introduction

Normal distribution is often used to describe the pattern of data in biomedical research [1–3]. Usually before conducting statistical analyses such as *t*-test, Hotelling *T*^2^ test and ANOVA, the normality test is performed. For example, in clinical trials, testing the effect of a new treatment is a basic problem, where two groups of subjects taking the treatment and placebo separately are enrolled and some features showing health benefits are screened. The *t*-test for univariate analysis and Hotelling *T*^2^ test for multivariate analysis are commonly employed to identify the mean differences between the two groups. However, the basic assumption of both tests is that data follow a normal distribution. If the normal distribution is violated, other nonparametric tests will be considered. It means that the results of the normality test may affect the inference of subsequent statistics. Another example is linear regression model, where the least square estimate may not be the best unbiased estimate when the error term does not obey the normal assumption. Futhermore, the inference of the regression coefficients based on *F*-test may be incorrect if the normality is violated.

For testing normality, the Quantile-Quantile (Q-Q) plot is a commonly used graphical method where the sample quantiles are compared with the expected ones from normal distribution. Generally, if the quantile points lie close to the diagonal line of the first and third quadrants, one can think of the data as being drawn from a normal distribution. A Percent-Percent plot can also be used based on two empirical cumulative distribution functions. Although both graphical methods are easy to operate, the decision depends on a rule of thumb. Thus the goodness-of-fit normality test were proposed [4]. Among many others, Shapiro and Wilk [5] developed a test (hereafter referred to as SW) considering the best linear unbiased estimate obtained by generalized least-squares method in the linear regression between sample order statistics and those for a standard normal distribution. SW is also regarded as squared Pearson correlation coefficient between ordered observations and some kind of weights. Since the original SW is limited to sample size 3 ≤ *n* ≤ 50, Royston [6] extended it to *n* ≤ 2000 and Rahman and Govidarajulu [7] extended to *n* ≤ 5000. Based on sample skewness and kurtosis, Jarque and Bera [8] proposed a test (hereafter referred to as JB). Tests based on the empirical distribution function *F*_*n*_ were also proposed, such as Kolmogorov-Smirnov test (hereafter referred to as KS), Cramér-Von Mises test (hereafter referred to as CVM), Anderson-Darling test (hereafter referred to as AD) [9]. KS is a supremum-type test to detect the difference between *F*_*n*_ and a specified normal distribution *F*_0_ with known mean and variance, and Lilliefors [10] improved KS by using sample mean and sample standard deviation to replace both unknown parameters, also commonly referred to as KS. CVM is a quadratic-type test and AD is the weighted version of CVM. In addition, there were also wide variety of normality tests corresponding to different types of alternative distributions in the literature. Dumonceaux et al. [11] considered the likelihood ratio test for some specific alternative distribution, such as Cauchy, exponential and double exponential. Spiegelhalter [12] gave the location and scale invariant test for uniform and double exponential distributions. Arshad et al. [13] proposed a modified AD for generalized Pareto distribution.

In view of the fact that there is no uniformly most powerful test under the general alternative hypothesis that data do not follow a normal distribution, we propose a Cauchy Combination Omnibus Test (CCOT). The CCOT aims to borrow the strength of AD, SW and JB, which is by far more robust than other tests under a wide range of settings. Another thrilling merit of the CCOT is that its statistical significance has an approximate expression, which is easier to calculate than other combination methods regardless of the correlation structure between the p-values.

The rest of the paper is structured as follows. In Section 2, we review some frequently-used methods, put forward the new test CCOT and give some theoretical results to discuss its effectiveness. Numerous simulation studies are shown in Section 3 and real applications are presented in Section 4. Some discussions are given in the last Section.

## Methods

### AD, SW and JB

Assume that *x*_1_, *x*_2_, ⋯, *x*_*n*_ *i*.*i*.*d*.∼*F*(*μ*, *σ*), where *μ* is the location parameter and *σ* is the scale parameter. The null hypothesis of testing problem is

$$\begin{array}{c} H_{0}:F\;\text{is}\;\text{a}\;\text{normal}\;\text{distribution}\;\text{function}, \end{array}$$

and the alternative hypothesis is

$$\begin{array}{c} H_{1}:F\;\text{is}\;\text{not}\;\text{a}\;\text{normal}\;\text{distribution}\;\text{function}. \end{array}$$

To test *H*_0_, Thadewald and Büning [14] set up a number of simulations for comparison among many tests and found that JB had higher power than others for symmetric distributions with medium up to long tails or slightly skewed distribution with long tails, and AD performed best for distributions with two peaks. Nornadiah and Yap [15] recommended SW for symmetric distributions with short tail or many skewed distributions. The advantages of AD, JB and SW cover a wide range of data distribution types, which have different lengths of tails, different degrees of skewness and different numbers of peaks, thus we mainly focus on these three tests here.

Considering the difference between the empirical distribution function and the normal distribution, Anderson and Darling [9] proposed a test as

$$\begin{array}{c} \text{AD}=n\int_{-\infty }^{\infty }\frac{{\left[F_{n}\left(x\right)-F_{0}\left(x\right)\right]}^{2}}{F_{0}\left(x\right)\left(1-F_{0}\left(x\right)\right)}\text{d}F_{0}\left(x\right), \end{array}$$

where *F*_0_(⋅) is the cumulative distribution of *F*(⋅) under *H*_0_ and $F_{n}\left(x\right)=\frac{1}{n}\sum_{i=1}^{n}I_{\left\{x_{i}\leq x\right\}}$. Given a nominal significance level *α*, *H*_0_ is rejected if the value of AD is larger than a threshold associated with *α* and sample size *n*. To make the threshold not dependent on the sample size *n*, Stephens [16] given the modified version as AD × (1 + 4/*n* − 25/*n*^2^).

Except for AD, there were two other similar tests: KS and CVM. AD is the weighted version of CVM by adding wight [*F*_0_(*x*)(1 − *F*_0_(*x*))]^−1^. Many simulation studies have shown that AD appears to have better performance than KS and CVM [14, 15, 17].

Shapiro and Wilk [5] proposed a test by using the ratio of two estimates for *σ*. We sort *x*_1_, *x*_2_, ⋯, *x*_*n*_ in an ascending order and denote them by *x*_(1)_, *x*_(2)_, ⋯, *x*_(*n*)_ with *x*_(1)_ ≤ *x*_(2)_ ≤ ⋯ ≤ *x*_(*n*)_. Let **x** = (*x*_(1)_, *x*_(2)_, …, *x*_(n)_)^⊤^ and *z*_1_, *z*_2_, ⋯, *z*_*n*_ be a random sample from a standard normal distribution *N*(0, 1) with ordered values being *z*_(1)_, *z*_(2)_, ⋯, *z*_(*n*)_, where *z*_(1)_ ≤ *z*_(2)_ ≤ ⋯ ≤ *z*_(*n*)_. Then

$$\begin{array}{c} x_{\left(i\right)}=\mu +\sigma z_{\left(i\right)}. \end{array}$$

Denote **z** = (*z*_(1)_, *z*_(2)_, …, *z*_(n)_)^⊤^, $u={\left(u_{1},u_{2},\ldots ,u_{n}\right)}^{⊤}={\left(Ez_{\left(1\right)},Ez_{\left(2\right)},\ldots ,Ez_{\left(n\right)}\right)}^{⊤}$ and *V* = (*v*_*ij*_)_*n* × *n*_ = (*cov*(*z*_(*i*)_, *z*_(*j*)_))_*n* × *n*_ as the mean vector and covariance matrix of order statistics, respectively. The best linear unbiased estimate of *σ* is (**u**^⊤^*V*^−1^**x**)/(**u**^⊤^*V*^-1^**u**). On the other hand ${\hat{\sigma }}^{2}=\frac{1}{n-1}\sum_{i=1}^{n}{\left(x_{i}-\bar{x}\right)}^{2}$ is the unbiased estimate of *σ*^2^. The SW was constructed as

$$\begin{array}{c} \text{SW}=\frac{{\left(w^{⊤}x\right)}^{2}}{\left(n-1\right){\hat{\sigma }}^{2}}=\frac{{\left(\sum_{i=1}^{n}w_{i}x_{\left(i\right)}\right)}^{2}}{\sum_{i=1}^{n}{\left(x_{i}-\bar{x}\right)}^{2}}, \end{array}$$

where **w** = (*w*_1_, *w*_2_, …, *w*_n_)^⊤^ = *V*^-1^**u**^⊤^/(**u**^⊤^*V*^-1^*V*^-1^**u**)^1/2^ and **w**^⊤^**w** = 1. Since the maximum value of SW is 1, SW will be close to 1 if *x*_1_, *x*_2_, ⋯, *x*_*n*_ are from a normal distribution. We reject the null hypothesis when SW is small. Since **w** is harder to calculate as the sample size *n* increases, SW is limited to sample size 3 ≤ *n* ≤ 50. Royston [6] extended SW to 4 ≤ *n* ≤ 2000 and Rahman and Govidarajulu [7] extended SW to *n* ≤ 5000. Moreover, Shapiro and Francia [18] gave another correlation type test (denoted by SF) by a new approximation to **w**’s.

Denote the sample skewness and kurtosis by

$$\begin{array}{c} b_{1}=\frac{\frac{1}{n}\sum_{i=1}^{n}{\left(x_{i}-\bar{x}\right)}^{3}}{{\left[\frac{1}{n}\sum_{i=1}^{n}{\left(x_{i}-\bar{x}\right)}^{2}\right]}^{3/2}},b_{2}=\frac{\frac{1}{n}\sum_{i=1}^{n}{\left(x_{i}-\bar{x}\right)}^{4}}{{\left[\frac{1}{n}\sum_{i=1}^{n}{\left(x_{i}-\bar{x}\right)}^{2}\right]}^{2}}. \end{array}$$

Since the skewness and kurtosis of a normal distribution are equal to to 0 and 3, respectively, Jarque and Bera [8] proposed a test by standardizing the sample skewness and kurtosis as

$$\begin{array}{c} \text{JB}=n[\frac{b_{1}^{2}}{6}+\frac{{\left(b_{2}-3\right)}^{2}}{24}]. \end{array}$$

Bowman and Shenton [19] showed that JB is the sum of squares of two asymptotically independent standard normal variables, thus JB follows $\chi_{2}^{2}$ asymptotically under *H*_0_ as *n* → ∞. Given a nominal significance level *α*, *H*_0_ is rejected if $\text{JB}>\chi_{2}^{2}\left(1-\alpha \right)$, where $\chi_{2}^{2}\left(1-\alpha \right)$ is the 1 − *α* quantile of *χ*^2^ distribution with 2 degrees of freedom.

### Cauchy combination omnibus test

Each of the above three test has its own sweet points: AD has outstanding performance for multimodal distributions, SW performs well for symmetric platykurtic with short-tailed distribution or skewed distributions, and JB is powerful for symmetric and slightly skewed distributions with long tails and poor for short-tailed distributions and bimodal distributions. Our goal is to construct a powerful and robust normality test that have relatively good performance over a wide range of situations. To borrow the strength from the above tests, we propose a cauchy combination omnibus test (CCOT) as

$$\begin{array}{c} \text{CCOT}=\frac{1}{3}\sum_{k=1}^{3}\text{tan}((0.5-p_{k})\pi ), \end{array}$$

where *p*_1_, *p*_2_ and *p*_3_ are the p-values of AD, SW and JB, respectively.

The reason we chose these three tests for the combination here is that their advantages cover most distributions, such as short tail to long tail distributions, symmetric to skewed distributions, unimodal or bimodal distributions.

With the available *p*_*k*_, *k* = 1, 2, 3, the statistical significance can be calculated based on the tail probability approximation via a standard Cauchy distribution [20–22]. Suppose that the observed value of CCOT is $\tilde{t}$, its approximate p-value is

$$\begin{array}{c} \text{p-value}\left(\tilde{t}\right)=\frac{1}{2}-\frac{\text{arctan}(\tilde{t})}{\pi }. \end{array}$$

**Remark 1**: Under *H*_0_, *p*_*k*_ ∼ *U*(0, 1) for *k* = 1, 2, 3, then

$$\begin{array}{c} \text{tan}((0.5-p_{k})\pi )∼Cauchy\left(0,1\right), \end{array}$$

where *Cauchy*(0, 1) is standard Cauchy distribution with the cumulative distribution function of $F_{CCOT}\left(t\right)=\frac{1}{2}+\frac{\text{arctan}(t)}{\pi }$. If *p*_1_, *p*_2_, *p*_3_ are independent of each other, $\text{CCOT}=\frac{1}{3}\sum_{k=1}^{3}\text{tan}((0.5-p_{k})\pi )∼Cauchy\left(0,1\right)$, and the p-value of CCOT is

$$\begin{array}{c} \text{p-value}\left(\tilde{t}\right)=P\left(CCOT>\tilde{t}|p_{1},p_{2},p_{3}\;iid\;∼U\left(0,1\right)\right)=1-F_{CCOT}\left(\tilde{t}\right)=\frac{1}{2}-\frac{\text{arctan}(\tilde{t})}{\pi }. \end{array}$$

On the other hand, if *p*_1_, *p*_2_, *p*_3_ are not independent of each other, its p-value can still be approximated by the above formula since the tail shape of the distribution of CCOT is similar to the tail of *Cauchy*(0, 1). The following numerical simulation result that CCOT can control the empirical type I error rate, shows the rationality of the p-value approximation.

The p-value of CCOT is between the minimum and maximum of three p-values *p*_1_, *p*_2_, *p*_3_. Thus, it is significant when *p*_1_, *p*_2_, *p*_3_ are all significant, and it is not significant when the smallest one is greater than the significance level. The corresponding conclusion can be referred to Chen [23]. However, in the normality test, we will encounter the situation where the results given by different methods are inconsistent, that is, some p-values in the combination are not significant. To explore how CCOT behaves in this situation, we consider the case of two p-values combination, where *p*_1_ is significant and *p*_2_ is not, and give the following Theorem 1.

**Theorem 1.** Denote $T=\frac{1}{2}\text{tan}((0.5-p_{1})\pi )+\frac{1}{2}\text{tan}((0.5-p_{2})\pi )$ and $p_{T}=\frac{1}{2}-\frac{\text{arctan}(T)}{\pi }$, where *p*_1_, *p*_2_ are two p-values and *p*_1_ ≤ *α*, *p*_2_ > *α*, *α* is a given level of significance, we have

(1) if *α* < *p*_2_ < 0.5, then *p*_*T*_ < *α* when *p*_2_ ∈ (*α*, 2*α* − *p*_1_];
(2) if 0.5 < *p*_2_ < 1, then
  (i) *p*_*T*_ < *α* when *p*_2_ ∈ (0.5, 0.5 + b*),
    where $b^{*}=\frac{1}{\pi }\text{arctan}[\text{tan}((0.5-p_{1})\pi )-2\;\text{tan}((0.5-\alpha )\pi )]>0$;
  (ii) $p_{T}>\frac{1}{2}$ when *p*_2_ ≥ 1 − *p*_1_.

**Remark 2**: Conclusion in (1) indicates that the p-value of *T* is significant as long as *p*_1_ + *p*_2_ ≤ 2*α*. For example, *p*_1_ = 0.01 and *α* = 0.05, *p*_*T*_ is significant when *p*_2_ ≤ 0.09. This result can also be generalized to combinations of more than two p-values, like $T=\frac{1}{m}\sum_{k=1}^{m}\text{tan}((0.5-p_{k})\pi )$, then *p*_*T*_ is significant as long as $\sum_{k=1}^{m}p_{k}\leq m\alpha$.

**Remark 3**: Conclusion in (i) of (2) indicates that the p-value of *T* is significant if *p*_1_ < *α* and *p*_2_ ∈ (0.5, 0.5 + b*). Here, b* is a number greater than 0, thus *p*_1_ needs to satisfy tan((0.5 − *p*_1_)*π*) > 2 tan((0.5 − *α*)*π*). For example, *α* = 0.05, then *p*_1_ ≤ 0.025155168. In particular, if *p*_1_ = 0.01, then b* = 0.48343 and *p*_*T*_ is significant as long as 0.5 < *p*_2_ < 0.98343. This also shows that when *p*_1_ is very small, *p*_2_ can have a larger range of values to cause the p-value of *T* to remain significant. In addition, conclusion in (ii) of (2) means that the p-value of *T* is not significant if *p*_1_ < *α* and *p*_2_ ≥ 1 − *p*_1_. If *p*_1_ = 0.01, $p_{T}>\frac{1}{2}$ when *p*_2_ ≥ 0.99. However, in the normality test, it is almost impossible for one method to have a very small p-value and the other method to have a p-value close to 1.

The detailed proof of Theorem 1 is as follows.

**Proof.** (1) If *p*_1_ ≤ *α* and *α* < *p*_2_ < 0.5, we have $0<((0.5-p_{k})\pi )<\frac{\pi }{2}$ for *k* = 1, 2. Since tan(⋅) is a convex function on interval $\left(0,\frac{\pi }{2}\right)$, according to Jensen inequality, we have

$$\begin{array}{c} T=\frac{1}{2}\text{tan}((0.5-p_{1})\pi )+\frac{1}{2}\text{tan}((0.5-p_{2})\pi )>\text{tan}((0.5-\frac{p_{1}+p_{2}}{2})\pi ). \end{array}$$

Then

$$\begin{array}{c} p_{T}=\frac{1}{2}-\frac{\text{arctan}(T)}{\pi }<\frac{1}{2}-\frac{\text{arctan}[\text{tan}((0.5-\frac{p_{1}+p_{2}}{2})\pi )]}{\pi }=\frac{p_{1}+p_{2}}{2}\leq \alpha , \end{array}$$

when *p*_2_ ≤ 2*α* − *p*_1_.

(2) If *p*_1_ ≤ *α* and *p*_2_ > 0.5, without loss of generality, let *p*_2_ = 0.5 + *b*, where *b* ∈ (0, 0.5).

Firstly, we prove the result of (ii). Since (0.5 − *p*_1_) > 0, (0.5−*p*_2_) < 0 and *p*_2_ ≥ 1 − *p*_1_, then *b* = (*p*_2_ − 0.5) ≥ (0.5 − *p*_1_) and we have

$$\begin{array}{c} \begin{array}{cc} T & =\frac{1}{2}\text{tan}((0.5-p_{1})\pi )+\frac{1}{2}\text{tan}((0.5-p_{2})\pi )\\ & =\frac{1}{2}[\text{tan}((0.5-p_{1})\pi )-\text{tan}((p_{2}-0.5)\pi )]\\ & =\frac{1}{2}[\text{tan}((0.5-p_{1})\pi )-\text{tan}(b\pi )]\\ & <0. \end{array} \end{array}$$

Thus $p_{T}=\frac{1}{2}-\frac{\text{arctan}(T)}{\pi }>\frac{1}{2}$.

Secondly, we prove the result of (i). Based on the conclusions of (i), *p*_*T*_ is not significant when *p*_2_ ≥ 1 − *p*_1_. Therefore, we consider 0.5 < *p*_2_ < 1 − *p*_1_, then 0 < *b* = (*p*_2_ − 0.5) < (0.5 − *p*_1_)<0.5. If $b^{*}=\frac{1}{\pi }\text{arctan}[\text{tan}((0.5-p_{1})\pi )-2\;\text{tan}((0.5-\alpha )\pi )]>0$, for *b* ∈ (0, b*), we have

$$\begin{array}{c} \begin{array}{cc} T & =\frac{1}{2}\text{tan}((0.5-p_{1})\pi )+\frac{1}{2}\text{tan}((0.5-p_{2})\pi )\\ & =\frac{1}{2}[\text{tan}((0.5-p_{1})\pi )-\text{tan}(b\pi )]\\ & >\frac{1}{2}[\text{tan}((0.5-p_{1})\pi )-\text{tan}(b^{*}\pi )]\\ & =\text{tan}((0.5-\alpha )\pi ). \end{array} \end{array}$$

Thus $p_{T}=\frac{1}{2}-\frac{\text{arctan}(T)}{\pi }<\frac{1}{2}-\frac{\text{arctan}[\text{tan}((0.5-\alpha )\pi )]}{\pi }=\alpha$.

Overall, the result of Theorem 1 shows that if *α* = 0.05 and *p*_1_ = 0.01, the p-value of the statistic based on the Cauchy combination can be guaranteed to be significant when *p*_2_ belongs to (0.05, 0.09] or (0.5, 0.98343). This also demonstates that CCOT is a robust and effective method in most cases, and the subsequent simulation results further support its good properties.

## Simulation studies

There is substantial simulation work in the literature to compare the power of normality tests by generating data from different distributions. Thadewald and Büning [14] generated data from the bimodal normal distribution (BN), which included symmetric distributions from short tail to long tail, asymmetric distributions and bimodal distributions. Nornadiah and Yap [15] carried out power comparisons of SW, KS and AD based on some symmetric or asymmetric familiar distributions. Following all of the above simulation settings, we conduct simulation studies by comparing the proposed CCOT with KS, CVM, AD, JB, SW, SF, and the traditional chi-square goodness-of-fit test (denoted by CS) that was given by Karl Pearson.

### The type I error rate

Assume that data are generated from *N*(0, 1) with

$$\begin{array}{c} n=10,15,20,25,30,40,50,100,200,300,400,500,1000,1500,2000. \end{array}$$

The number of replicates for calculating the empirical type I error rates is 10,000. Table 1 presents the empirical type I error rates of eight tests. From Table 1, except JB, the type I error rate of CCOT and other tests are all close to 0.05. JB is a little conservative because it has inaccurate chi-square approximation for small samples due to slow convergence of kurtosis, but this phenomenon is improved when the sample size is large. Take *n* = 30, 50, 200, 500, 1500 for example, the empirical type I error rates of CCOT are 0.0456, 0.0526, 0.0501, 0.0553, and 0.0549, respectively, while JB gives 0.0313, 0.0357, 0.0444, 0.0472, and 0.0519, respectively.

**Table 1:** The empirical type I error rates of eight tests for different sample sizes under nominal significance level 0.05 based on 10,000 replications.

*n*	CCOT	KS	CVM	AD	JB	SW	SF	CS
10	0.0364	0.0544	0.0520	0.0531	0.0088	0.0521	0.0555	0.0704
15	0.0371	0.0482	0.0470	0.0469	0.0179	0.0458	0.0492	0.0511
20	0.0433	0.0492	0.0496	0.0496	0.0225	0.0499	0.0522	0.0489
25	0.0474	0.0502	0.0510	0.0520	0.0249	0.0518	0.0519	0.0626
30	0.0456	0.0536	0.0488	0.0482	0.0313	0.0485	0.0525	0.0531
40	0.0466	0.0490	0.0512	0.0495	0.0329	0.0492	0.0506	0.0621
50	0.0526	0.0502	0.0521	0.0494	0.0357	0.0493	0.0510	0.0490
100	0.0542	0.0482	0.0491	0.0493	0.0451	0.0483	0.0529	0.0494
200	0.0501	0.0497	0.0457	0.0460	0.0444	0.0487	0.0509	0.0496
300	0.0569	0.0491	0.0491	0.0490	0.0474	0.0496	0.0526	0.0504
400	0.0517	0.0494	0.0480	0.0480	0.0433	0.0476	0.0468	0.0527
500	0.0553	0.0512	0.0477	0.0495	0.0472	0.0508	0.0530	0.0551
1000	0.0566	0.0473	0.0507	0.0523	0.0491	0.0519	0.0541	0.0533
1500	0.0549	0.0461	0.0515	0.0515	0.0519	0.0517	0.0531	0.0502
2000	0.0483	0.0454	0.0444	0.0441	0.0476	0.0468	0.0496	0.0504

### Bimodal normal distribution

Assume that data are generated from the bimodal normal distribution (BN):

$$\begin{array}{c} \left(1-a\right)N\left(\mu_{1},\sigma_{1}^{2}\right)+aN\left(\mu_{2},\sigma_{2}^{2}\right), \end{array}$$

where *a* ∈ [0, 1] means that data come from $N(\mu_{2},\sigma_{2}^{2})$ with probability of *a*, and from $N(\mu_{1},\sigma_{1}^{2})$ with probability of 1 − *a*. For convenience, we fix *μ*_1_ = 0, $\sigma_{1}^{2}=1$ and choose the nominal significance level *α* = 0.05. The number of replicates for calculating the empirical power is 10,000. We choose *μ*_2_ ∈ {0, 1, 2, 3, 4}, *σ*_2_ ∈ {1, 2, 3, 4, 6} and *a* ∈ {0.01, 0.05, 0.20, 0.35, 0.50, 0.65, 0.80, 0.90, 0.95, 0.99}. The combination of these parameters allows for a variety of distributions, such as symmetric and asymmetric cases, unimodal and bimodal cases. We set five scenarios: (i) *μ*_2_ = 0, *a* = 0.1, *n* = 100 and (ii) *μ*_2_ = 0, *a* = 0.5, *n* = 100 for different *σ*_2_, (iii) *σ*_2_ = 4, *a* = 0.05, *n* = 50 and (iv) *σ*_2_ = 1, *a* = 0.5, *n* = 50 for different *μ*_2_, and (v) *μ*_2_ = 0, *σ*_2_ = 4, *n* = 50 for different *a*. Scenario (i) corresponds to symmetric distributions with moderate to long tails; Scenario (ii) corresponds to symmetric distributions that data come from $N(\mu_{1},\sigma_{1}^{2})$ and $N(\mu_{2},\sigma_{2}^{2})$ with equal probability; Scenario (iii) corresponds to slightly skewed distribution with long tails; Scenario (iv) corresponds to the distributions with two peaks; and Scenario (v) focuses on the effect of different probabilities of *a*. Their density function curves, skewness and kurtosis are presented in S1 Fig.

**Fig 1:**
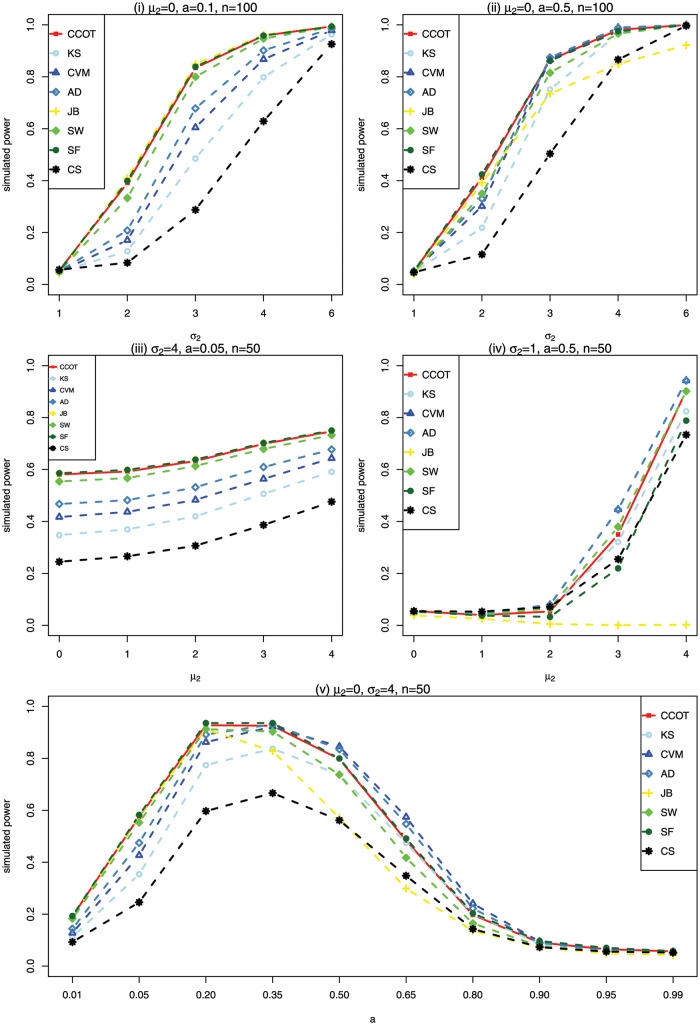
The empirical power for scenarios (i)—(v) under nominal significance level 0.05 based on 10,000 replications.

Fig 1 illustrates the empirical power of CCOT and other tests for Scenarios (i)—(v). We can see from Fig 1 that under Scenario (i), CCOT, JB and SF perform better than SW, AD, CVM, KS, and CS. Taking *σ*_2_ = 3 of Scenario (i) for an example, the powers of CCOT, JB, SF, SW, AD, CVM, KS, CS are 0.8343, 0.8458, 0.8387, 0.7998, 0.6782, 0.6044, 0.4850, 0.2874, respectively. Under Scenario (ii), the empirical power of AD and CVM goes up while JB’s goes down, meanwhile CCOT has a good performance follows AD and CVM. Under Scenario (iii), CCOT, JB and SF are superior to other methods. Taking *μ*_2_ = 2 of Scenario (iii) as an example, the powers of CCOT, JB, SF, KS, CVM AD, SW, CS are 0.6327, 0.6385, 0.6381, 0.4198, 0.4829, 0.5315, 0.6134, 0.3065, respectively. Under Scenario (iv), CCOT, CVM, AD and SW have higher power than other tests. Taking *μ*_2_ = 4 of Scenario (iv) as an example, the powers of CCOT, CVM, AD, SW, KS, JB, SF, CS are 0.9032, 0.9430, 0.9425, 0.9017, 0.8241, 0.0018, 0.7884, 0.7340, respectively. Under Scenario (v), as probability *a* increases, JB performs well only at high kurtosis and poorly at low kurtosis, AD and CVM perform better when 0.35 ≤ *a* ≤ 0.8, while the proposed CCOT is robust in all *a*’s. Taking *a* = 0.35 as an example, the powers of CCOT, CVM, AD, JB, SW, CS are 0.9257, 0.9224, 0.9317, 0.8283, 0.9034, 0.6666, respectively.

### Other distributions

Let *n* = {10, 15, 20, 25, 30, 40, 50, 100, 200, 300, 400, 500, 1000, 1500, 2000} and the nominal significance level is chosen as *α* = 0.05. For comparison of power, we generate data from 12 common non-normal distributions consisting of 6 symmetric distributions:

$$\begin{array}{c} \text{Beta}\left(2,2\right),\;\;t_{300},\;\;t_{10},\;\;t_{7},\;\;\text{Laplace}\left(0,1\right),\;\;t_{5}, \end{array}$$

and 6 asymmetric distributions:

$$\begin{array}{c} \text{Beta}\left(6,2\right),\;\;\text{Beta}\left(3,2\right),\;\;\chi_{20}^{2},\;\;\text{Gamma}\left(4,5\right),\;\;\chi_{4}^{2},\;\;\text{Gamma}\left(1,5\right). \end{array}$$

**Fig 2:**
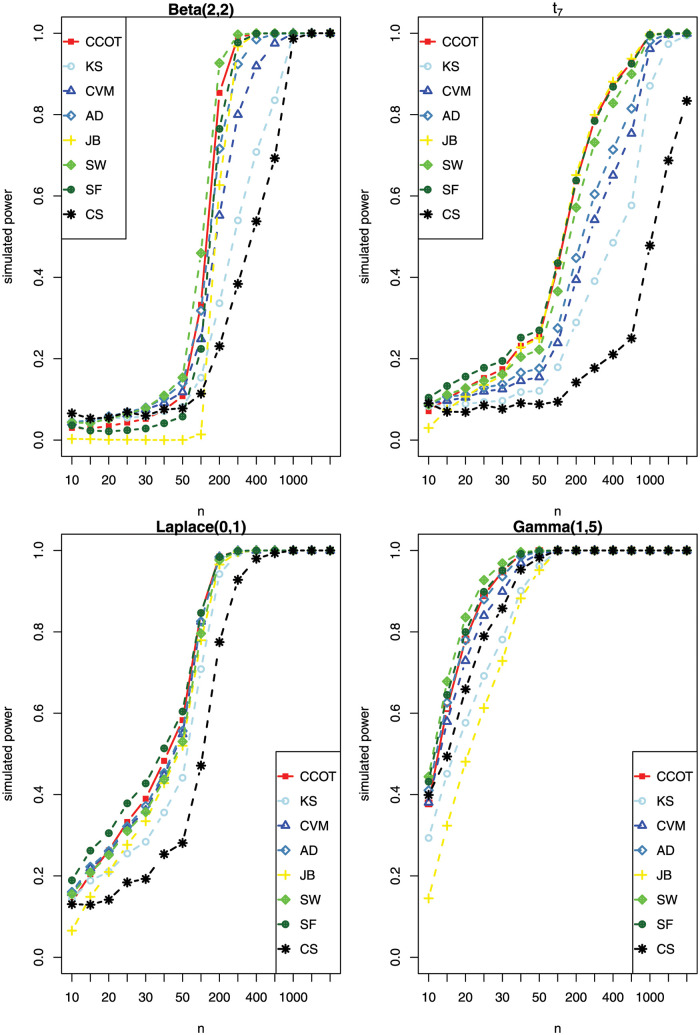
The empirical power for different sample sizes under nominal significance level 0.05 based on 10,000 replications.

The density function curves, skewness and kurtosis of these 12 non-normal distributions are displayed in S2 Fig. The calculation of empirical power is based on 10,000 replications. For convenience, we present some representative results as shown in Fig 2, and the remaining scenarios are presented in S3 Fig. Beta(2; 2) correspond to the symmetrical platykurtic distributions with short tail, *t*_7_ and Laplace(0,1) correspond to the symmetrical leptokurtic distributions with medium long tail, Gamma(1,5) corresponds to asymmetrical distribution.

Fig 2 gives the empirical power of 4 common non-normal distributions. As depicted in Fig 2, for Beta(2; 2), CCOT and SW are the two most powerful tests. Taking *n* = 200 for an example, the powers of CCOT, SW, KS, CVM, AD, JB, SW, CS are 0.8535, 0.9267, 0.3364, 0.5521, 0.7163, 0.6267, 0.765, 0.2307, respectively. For *t*_7_ and Laplace(0,1), CCOT, JB and SF have higher power than other tests. Taking *n* = 300 for *t*_7_ as an example, the powers of CCOT, JB, SF, KS, CVM, AD, SW, CS are 0.7879, 0.7995, 0.7843, 0.3907, 0.5414, 0.6043, 0.7317, 0.1770, respectively. For Gamma(1,5), the power of CCOT, SW and SF are higher than other tests. For example, when *n* = 20, CCOT, SW and SF give power of 0.7814, 0.8356 and 0.7995, and KS, CVM, AD, JB, and CS give power of 0.5765, 0.7288, 0.7796, 0.4808, and 0.6589, respectively. Overall, CCOT is a powerful and robust test when data come from different distributions. The other results in S3 Fig also show the robustness and efficiency of CCOT in different scenarios.

## Applications

### Application to South African heart disease data

The Coronary Risk-Factor Study (CORIS) was jointly initiated in 1978 by the South African Medical Research Council, the Department of Health, and Welfare and Human Sciences Research Council. The aim is to determine the prevalence and intensity of risk factors in an Afrikaner community and assess the effectiveness of interventions to reduce risk factors. Rousseauw et al. [24] used the data of CORIS in three rural communities of the southwestern Cape Province to identify and establish the intensity of ischaemic heart disease (IHD) risk factors. A subset data of Rousseauw et al. [24] were analyzed by Hastie and Tibshirani [25] to study the risk factors for myocardial infarction, consisting of 462 white males with 162 patients and 302 healthy people between ages 15 and 64. The subset data include quantitative indicators of 9 risk factors: systolic blood pressure, cumulative tobacco, low density lipoprotein cholesterol (LDL), adiposity, family history of heart disease, type-A behavior (TYPE-A), obesity, current alcohol consumption, and age at onset (AGE). Our analysis below is based on this subset data that can be found in the ‘SAheart’ of R-package “ElemStatLearn”. For convenience, we select three risk factors (LDL, TYPE-A, AGE) to test whether the corresponding patient group and healthy control group come from normal distribution. We apply CCOT and other representative normality tests to the data set and obtain their p-values as shown in Table 2.

**Table 2:** The p-values of normality tests for South African heart disease data.

Factors	Groups	*n*	CCOT	KS	CVM	AD	JB	SW	SF
LDL	patient	160	2.2 × 10^−11^	5.2 × 10^−4^	6.6 × 10^−6^	3.2 × 10^−7^	7.2 × 10^−12^	3.9 × 10^−7^	1.8 × 10^−6^
	control	302	0	9.6 × 10^−7^	2.8 × 10^−8^	2.4 × 10^−11^	0	6.2 × 10^−12^	9.4 × 10^−11^
TYPE-A	patient	160	7.6 × 10^−1^	9.2 × 10^−1^	9.3 × 10^−1^	8.9 × 10^−1^	5.8 × 10^−1^	5.7 × 10^−1^	4.6 × 10^−1^
	control	302	5.8 × 10^−4^	3.4 × 10^−2^	5.5 × 10^−2^	2.3 × 10^−2^	2.1 × 10^−4^	2.9 × 10^−3^	2.4 × 10^−3^
AGE	patient	160	3.6 × 10^−9^	3.2 × 10^−7^	1.4 × 10^−7^	1.2 × 10^−9^	1.1 × 10^−4^	1.5 × 10^−7^	1.3 × 10^−6^
	control	302	2.3 × 10^−9^	2.3 × 10^−4^	5.2 × 10^−6^	9.9 × 10^−10^	1.3 × 10^−4^	3.6 × 10^−9^	7.8 × 10^−8^

We can see from the Table 2 that except for TYPE-A in the patient group, other scenarios all show that the null hypothesis should be rejected at the nominal significance level of 0.05. For risk factor LDL, the proposed CCOT and JB have the smaller p-values than those of AD and SW. Taking LDL in the patient group as example, the p-values of CCOT and JB are 2.2 × 10^−11^ and 7.2 × 10^−12^, while KS, CVM, AD, SW, and SF give 5.2 × 10^−4^, 6.6 × 10^−6^, 3.2 × 10^−7^, 3.9 × 10^−7^ and 1.8 × 10^−6^, respectively. For risk factor TYPE-A, it is obvious that the samples from patient group are normally distributed. For TYPE-A in the patient group, CCOT has a larger p-value than JB, SW and SF. The p-values of CCOT and AD are 0.76 and 0.89, while JB, SW and SF give 0.58, 0.57 and 0.46, respectively. For TYPE-A in the control group, the p-values of all tests but CVM are less than 0.05 and CCOT and JB have smaller p-values than other tests. For example, the p-values of CCOT and JB are 5.8 × 10^−4^ and 2.1 × 10^−4^, while KS, CVM, AD, SW, and SF give 3.4 × 10^−2^, 5.5 × 10^−2^, 2.3 × 10^−2^, 2.9 × 10^−3^, and 2.4 × 10^−3^, respectively. For risk factor AGE, the p-values of CCOT and AD are the smallest in patient group. For example, for AGE in the patient group, the p-values of CCOT and AD are 3.6 × 10^−9^ and 1.2 × 10^−9^, while KS, CVM, JB, SW, and SF give 3.2 × 10^−7^, 1.4 × 10^−7^, 1.1 × 10^−4^, 1.5 × 10^−7^, and 1.3 × 10^−6^, respectively.

### Application to neonatal hearing impairment data

Norton et al. [26] considered three neonatal hearing screening tools: distortion product otoacoustic emissions (DPOAE), transient evoked otoacoustic emissions (TEOAE) and auditory brain stem responses (ABR) to identify neonatal hearing impairment. The three tools were usually regarded as three biomarkers in the field of biomedical sciences. Taking female data as example, it includes 2,234 samples that are collected from one ear or two ears (left ear and right ear) of infants, where there are 64 hearing impaired samples (patient group) and 2,170 normal samples (control group). Statistical analysis often focuses on whether there are some differences between the two groups in different biomarkers. *t*-test for the means of two groups is a simple and common parametric method to solve this issue, but the premise is that levels of the biomarker come from normal distributions.

**Fig 3:**
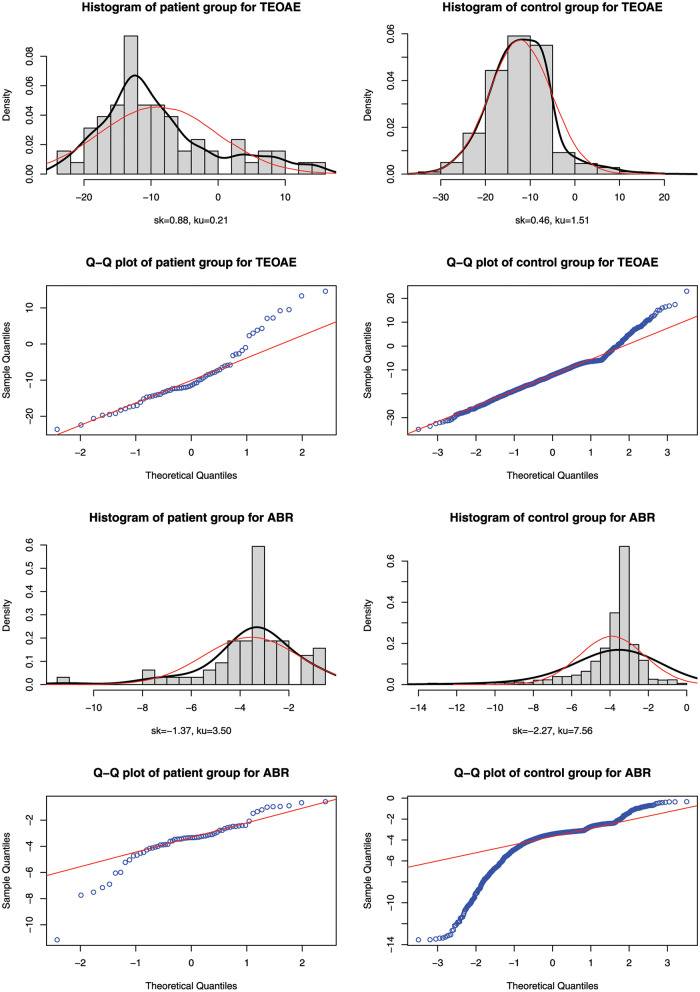
The Q-Q plots of patient group and health group for biomarkers TEOAE and ABR.

Choosing two biomarkers TEOAE and ABR as examples, the Q-Q plots of patient group and control group are presented in Fig 3. Deviation from the straight lines of Q-Q plots in Fig 3 indicates that both biomarkers in each group do not seem to follow normal distributions. We apply CCOT and other normality tests to this data and calculate their p-values as shown in Table 3. All methods reject the null hypothesis at the nominal significance level of 0.05. For biomarker TEOAE, CCOT, AD and CVM have the lowest p-values in patient group. For example, the p-values of CCOT, AD and CVM for patient group are 6.0 × 10^−4^, 2.5 × 10^−4^ and 3.3 × 10^−4^, while KS, JB, SW and SF give 1.1 × 10^−2^, 1.1 × 10^−2^, 1.1 × 10^−3^, and 2.2 × 10^−3^, respectively. For biomarker ABR, the p-values of CCOT and JB are samller than other tests in patient group. For example, the p-values of CCOT and JB are 8.7 × 10^−13^ and 2.9 × 10^−13^, while KS, CVM, AD, SW, and SF give 1.1 × 10^−3^, 4.5 × 10^−5^, 3.4 × 10^−5^, 2.8 × 10^−5^, and 4.9 × 10^−5^, respectively. For control group with larger sample size, all normality tests have very small p-values and CCOT also follows this trend. Taking ABR in the control group as example, both CCOT and JB have p-values of 0, and KS, CVM, AD, SW, and SF give p-values of 3.4 × 10^−209^, 7.4 × 10^−10^, 3.7 × 10^−24^, 1.3 × 10^−46^, and 1.6 × 10^−42^, respectively. These results also confirm with Q-Q plots that data deviate from the normal distributions. Thus *t*-test cannot be used to detect the differences between the two groups, whereas nonparametric methods such as Wilcoxon rank-sum test should be considered.

**Table 3:** The p-values of normality tests for neonatal hearing impairment female data.

Biomarkers	Groups	*n*	CCOT	KS	CVM	AD	JB	SW	SF
TEOAE	patient	64	6.0 × 10^−4^	1.1 × 10^−2^	3.3 × 10^−4^	2.5 × 10^−4^	1.1 × 10^−2^	1.1 × 10^−3^	2.2 × 10^−3^
	control	2170	0	2.8 × 10^−42^	7.4 × 10^−10^	9.2 × 10^−23^	0	2.5 × 10^−18^	4.1 × 10^−17^
ABR	patient	64	8.7 × 10^−13^	1.1 × 10^−3^	4.5 × 10^−5^	3.4 × 10^−5^	2.9 × 10^−13^	2.8 × 10^−5^	4.9 × 10^−5^
	control	2170	0	3.4 × 10^−209^	7.4 × 10^−10^	3.7 × 10^−24^	0	1.3 × 10^−46^	1.6 × 10^−42^

## Discussion

Prior to applying a normality-based statistical inference procedure, it is critical to check whether the data come from a normal distribution. The South African Heart Disease and Neonatal Hearing Impairment data all involve the testing problem of whether there are some differences between patient and healthy groups. It is related to *t*-test or Hotelling *T*^2^, while the precondition of both tests is that data should be are normal- or approximately normal-distributed. Thus, performing normality test before data analyses is helpful to understand the data structure and verify the effect of further statistical analysis.

In this article, we review some representative and commonly-used normality tests such as AD, SW and JB. In addition to these three tests, other normality tests that have some merits can also be taken into account, but for simplicity we have chosen only the three most representative ones. Since AD, SW and JB are constructed by the distribution shape, second moment, and the third and fourth moments, they are widely applied in applications. Based on numerical results, we find that each of them has the sweet point: AD has outstanding performance for multimodal distributions, SW performs well for symmetric platykurtic with short-tailed distribution or skewed distributions, and JB is powerful for symmetric and slightly skewed distributions with long tails and poor for short-tailed distributions and bimodal distributions. However, the data in the actual problem may be complex and diverse, and the shape of its distribution may show different degrees of skewness and kurtosis, or it may show multi-peak state. Thus, a new test called CCOT is proposed by integrating the superiorities of the above three tests, which can be applied to different data distributions. We take two p-value combinations as an example in Theorem 1 to discuss the conditions under which CCOT remains significant when one p-value in the combination is significant but the other p-value is not. For example, if *α* = 0.05 and *p*_1_ = 0.01, the p-value of the statistic based on the Cauchy combination can be guaranteed to be significant when *p*_2_ ∈ (0.05, 0.09] or (0.5, 0.98343). Besides, the p-value of *T* is not significant if *p*_1_ = 0.01 and *p*_2_ ≥ 0.99, but it is very rare for the p-values of the two methods to differ greatly in the normality test.

There are a variety of combined p-value strategies in many literatures based on different research purposes and data characteristics, such as minimum p-value method [27], Fisher’s combination test [28], higher criticism method [29], adaptive rank truncated product method [30], Cauchy combination test [21], and so on. However, no method is uniformly most powerful in all cases [31, 32]. In this paper, the CCOT has two advantages: (i) its performance is always close to the best method in numerical simulation, (ii) its statistical significance has an approximate expression, which is easier to calculate than other combination methods regardless of the correlation structure between the p-values. We also give the source R code to compute its p-value, which is attached in S1 Appendix. The result of case analysis shows that CCOT is more robust and effective because it can detect the difference from the normal distribution to serve the subsequent selection of appropriate statistical inference methods. Thus it is expected that CCOT has wide useful application in the future.

In this work, we focus on univariate normal test. There are some work directly extending the procedures of univariate normality test to multivariate cases. Mardia [33] gave sample estimates of multivariate skewness or kurtosis, which provided a reference for multivariate normality test based on sample moments. Kim [34] proposed a multivariate version of the Jarque-Bera test using orthogonalization or empirical standardization of data, which was powerful for symmetric marginal distributions with long tails. Shapiro and Wilk test has been generalized to multivariate cases [35, 36]. For multivariate normality test based on empirical distribution function, a common idea is to reduce the multivariate data to univariate uniformity [37]. It’s worth noting that our Cauchy combination omnibus test can be naturally extended to the multivariate normality test because it just needs the p-values.

## Supporting information

S1 Fig The density function curves for Scenarios (i)—(v) under BN model. The solid and dashed lines represent the density function curves of standardized BN samples and standard normally distributed samples, respectively. The values of sk and ku below these subfigures are the skewness and kurtosis.
(PDF)

S2 Fig The density function curves, skewness and kurtosis of 12 common non-normal distributions. The solid and dashed lines represent the density curves of 12 common non-normal distributed data and standard normally distributed data, respectively.
(PDF)

S3 Fig The empirical power for different sample sizes under remaining 8 common non-normal distributions at nominal significance level of 0.05 based on 10,000 replications. (PDF)

S1 Appendix The source R code to compute p-value of CCOT. (PDF)
