# Supplementary figures and images for: Cauchy combination omnibus test for normality
Source: PLoS One. 2023 Aug 3;18(8):e0289498. doi: 10.1371/journal.pone.0289498 (PMC10399863; doi:10.1371/journal.pone.0289498)

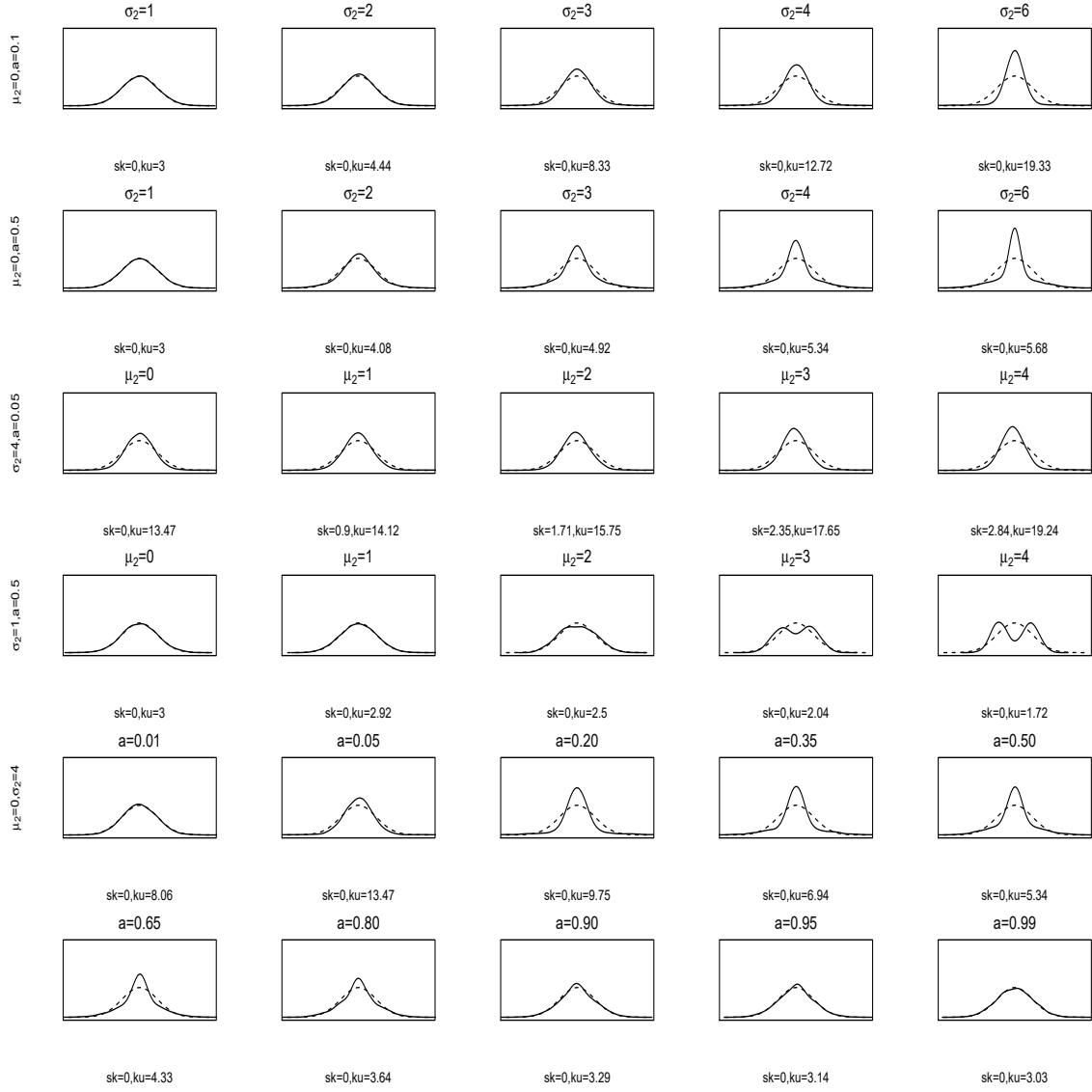

Supplement: S1 Fig — The solid and dashed lines represent the density function curves of standardized BN samples and standard normally distributed samples, respectively. The values of sk and ku below these subfigures are the skewness and kurtosis. (PDF) [file pone.0289498.s001.pdf]

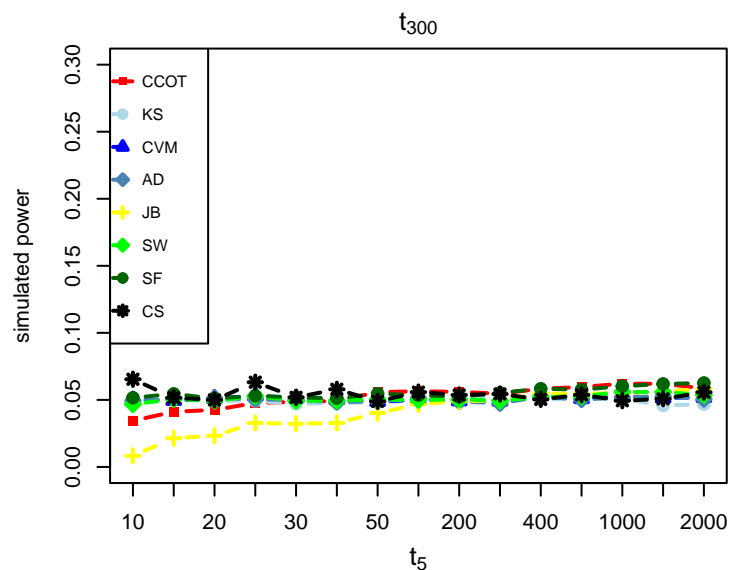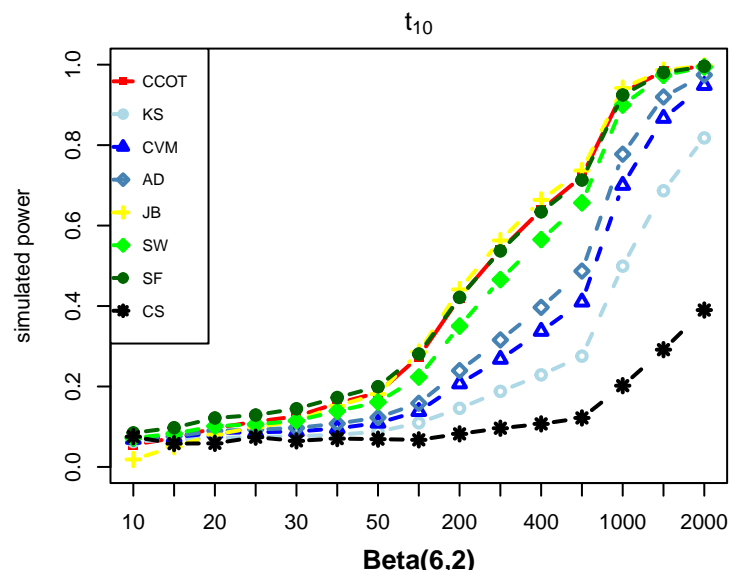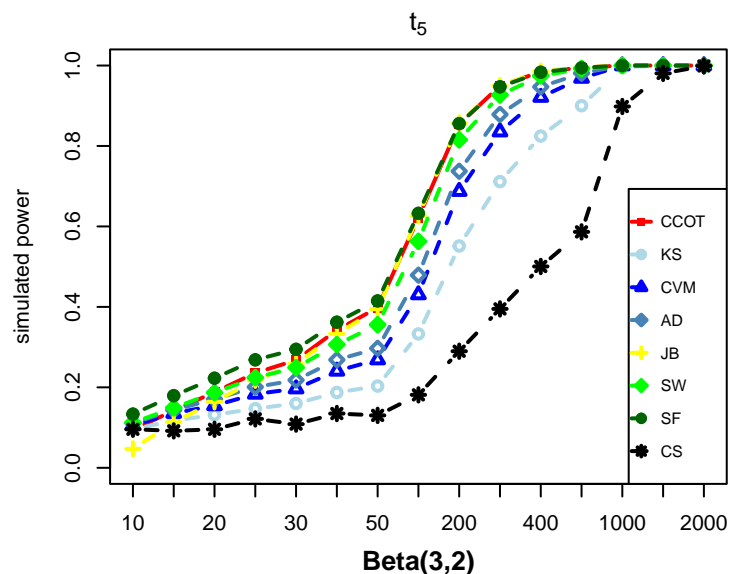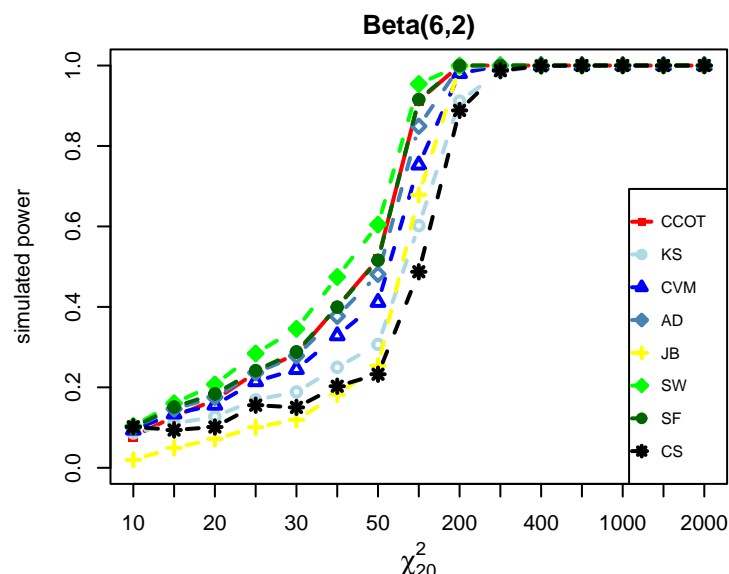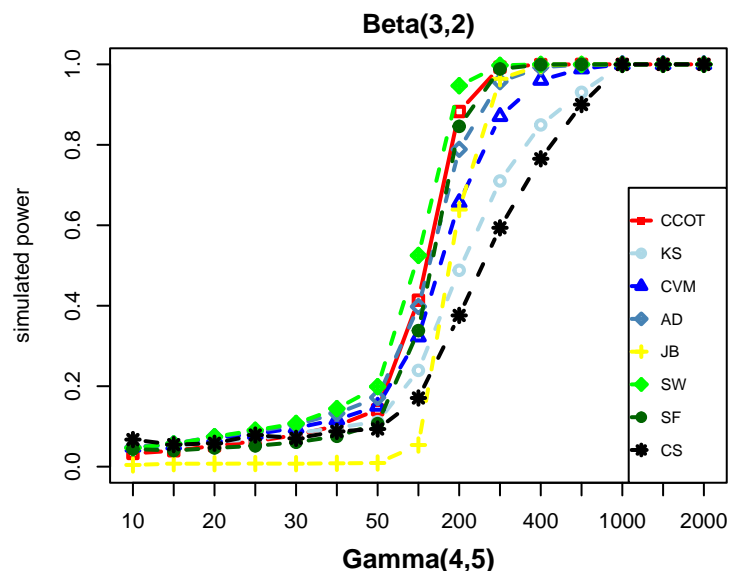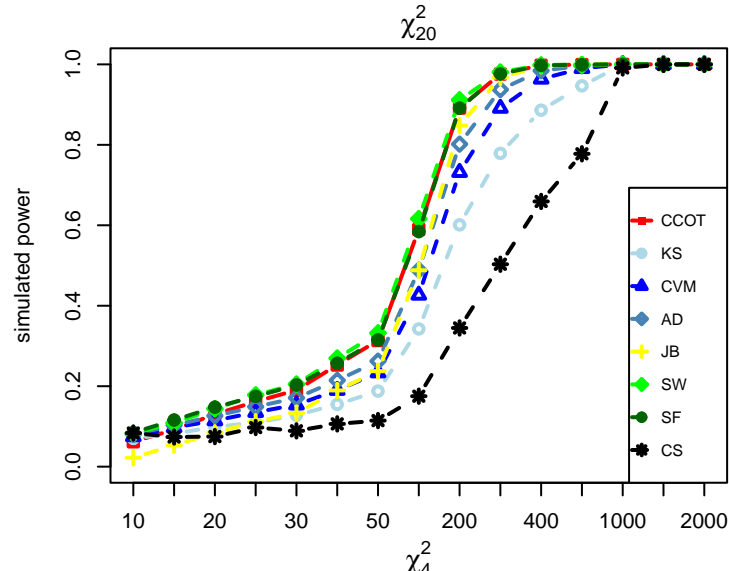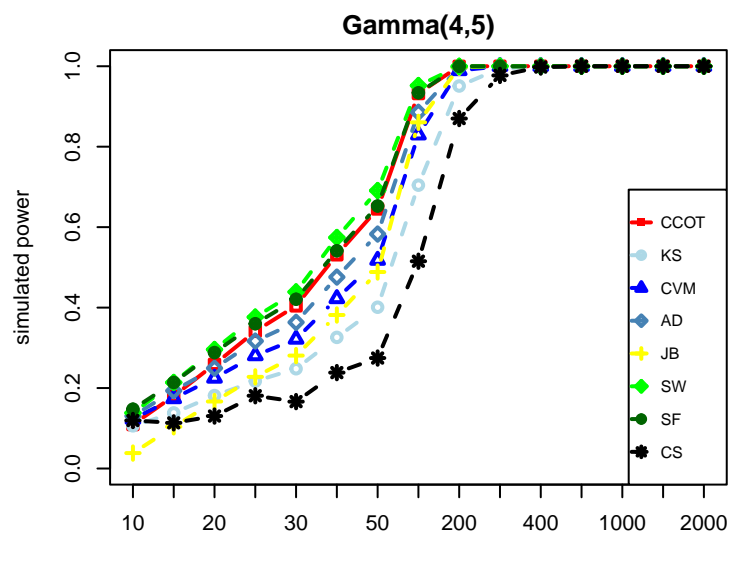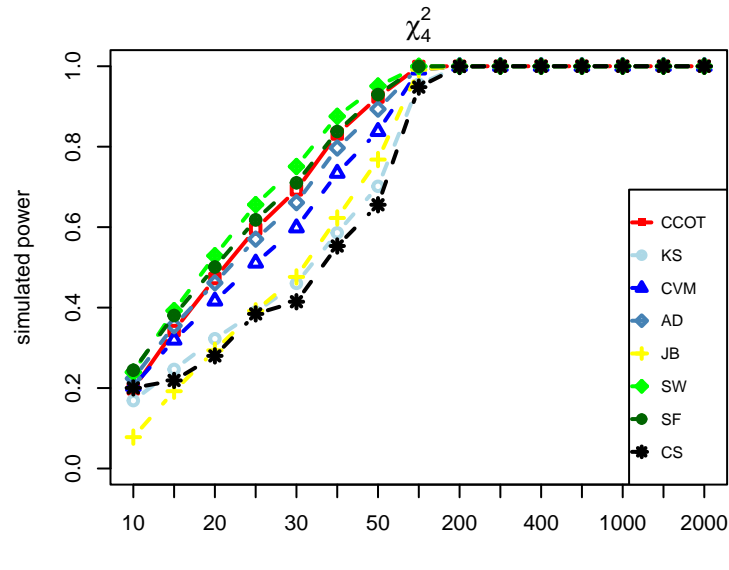

Supplement: S3 Fig — (PDF) [file pone.0289498.s003.pdf]
