# Supplementary material for: Cauchy combination omnibus test for normality
Source: PLoS One. 2023 Aug 3;18(8):e0289498. doi: 10.1371/journal.pone.0289498 (PMC10399863; doi:10.1371/journal.pone.0289498)
Supplement: S2 Fig — The solid and dashed lines represent the density curves of 12 common non-normal distributed data and standard normally distributed data, respectively. (PDF) [file pone.0289498.s002.pdf]

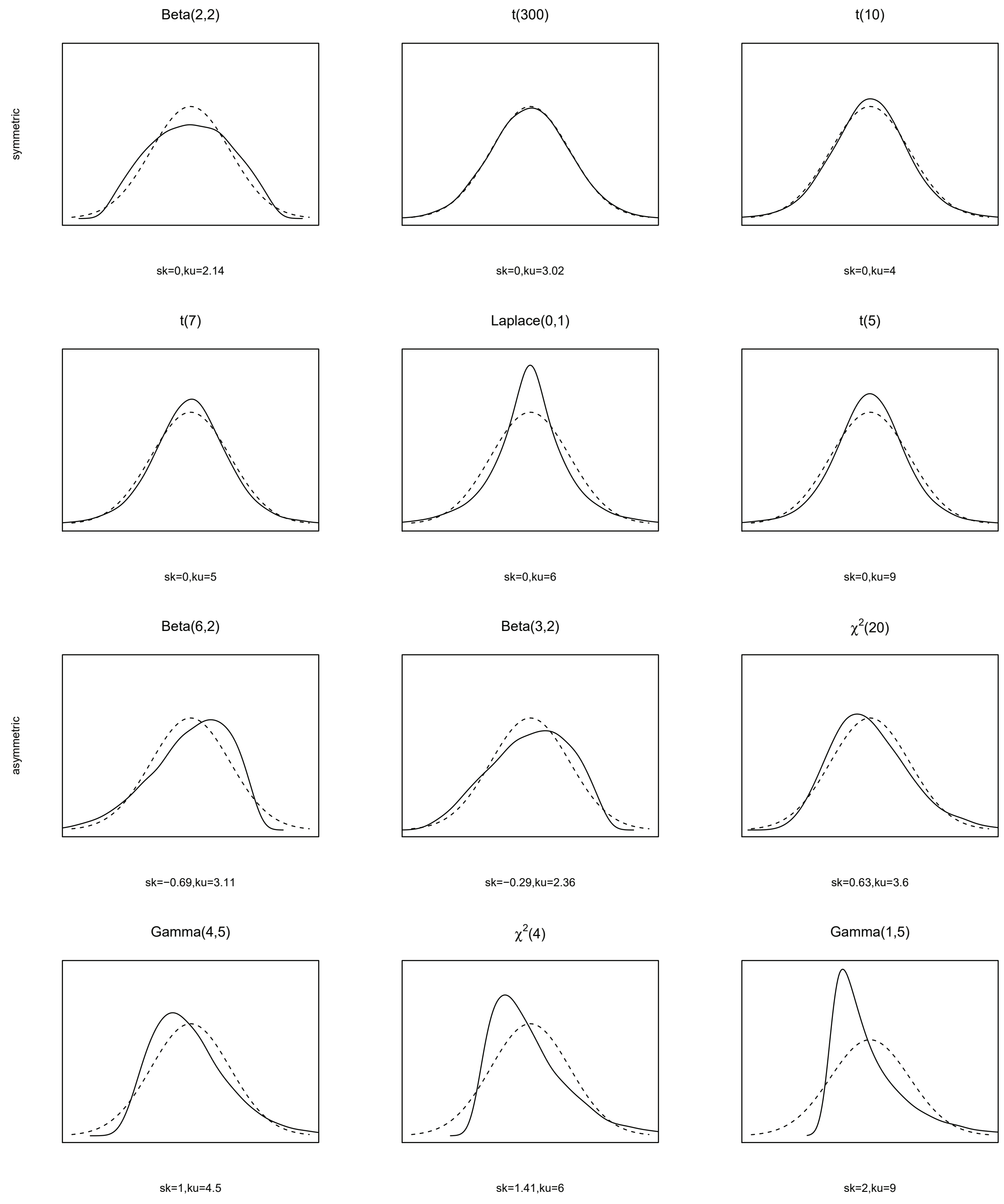
symmetric
asymmetric
Beta(2,2)
sk=0,ku=2.14
t(300)
sk=0,ku=3.02
t(10)
sk=0,ku=4
t(7)
sk=0,ku=5
Laplace(0,1)
sk=0,ku=6
t(5)
sk=0,ku=9
Beta(6,2)
sk=−0.69,ku=3.11
Beta(3,2)
sk=−0.29,ku=2.36
χ2(20)
sk=0.63,ku=3.6
Gamma(4,5)
sk=1,ku=4.5
χ2(4)
sk=1.41,ku=6
Gamma(1,5)
sk=2,ku=9
