# Supplementary material for: Cauchy combination omnibus test for normality
Source: PLoS One. 2023 Aug 3;18(8):e0289498. doi: 10.1371/journal.pone.0289498 (PMC10399863; doi:10.1371/journal.pone.0289498)
Supplement: S1 Appendix — (PDF) [file pone.0289498.s004.pdf]

```
CAL.pval <- function(X)
{
  n <- length(X)
  #Anderson-Darling test
  pval.AD <- ad.test(X)$p.value
  #Jarque-Bera test
  pval.JB <- jarque.test(X)$p.value
  #Shapiro-Wilk test
  pval.SW <- shapiro.test(X)$p.value

  #Cauchy Combination Omnibus Test (CCOT)
  tilde.p <- c(pval.AD, pval.SW, pval.JB)
  T.c <- mean(tan((0.5-tilde.p)*pi))
  pval.CCOT <- 1/2 - atan(T.c)/pi
  pval.CCOT
}
```
